# Counteracting Grey Mould (*Botrytis cinerea*) in Grapevine ‘Glera’ Using Three Putative Biological Control Agent Strains (*Paraburkholderia* sp., *Pseudomonas* sp., and *Acinetobacter* sp.): Impact on Symptoms, Yield, and Gene Expression

**DOI:** 10.3390/microorganisms12081515

**Published:** 2024-07-24

**Authors:** Giovanni Mian, Nicola Belfiore, Patrick Marcuzzo, Francesco Spinelli, Diego Tomasi, Andrea Colautti

**Affiliations:** 1 Department of Agricultural and Food Sciences, Alma Mater Studiorum Università di Bologna, 40129 Bologna, Italy; giovanni.mian2@unibo.it (G.M.); francesco.spinelli3@unibo.it (F.S.); 2 Council for Agricultural and Economics-Research-Centre for Viticulture and Oenology, Viale Aprile, 26, 31015 Conegliano, Italy; nicola.belfiore@crea.gov.it (N.B.); patrick.marcuzzo@crea.gov.it (P.M.); 3 Consorzio Tutela del Vino Conegliano Valdobbiadene Prosecco, Piazza Libertà, 7, 31053 Pieve di Soligo, Italy; diego.tomasi@prosecco.it; 4 Department of Agricultural, Food, Environmental and Animal Science (Di4A), University of Udine, 33100 Udine, Italy

**Keywords:** biological control agents, *Vitis vinifera*, *Paraburkholderia* spp., *Pseudomonas* spp., *Acinetobacter* spp., *Botrytis cinerea*, gene expression

## Abstract

This study examined the potential use of three bacterial strains—*Paraburkholderia* sp. strain CRV74, *Pseudomonas* sp. strain CRV21, and *Acinetobacter* sp. strain CRV19—as biocontrol agents of *Botrytis cinerea* in grapevine. These strains were selected for their ability to inhibit *B. cinerea* growth in vitro and used in field conditions for the control of grey mould symptoms in ‘Glera’ grapes. To this end, after inoculating these microorganisms onto plants sprayed with *B. cinerea* spores, the final yield, the physicochemical characteristics of the must, disease incidence, and the possible influence on the expression of plant-defence proteins were evaluated. Strain CRV21 resulted as being the most effective in combating grey mould (−20% of disease incidence). Although yield was not affected, significantly different values of total soluble solids content was observed. Additionally, a significant up-regulation of the genes PR-1, PR-5, β-1,3-glucanase, and class III chitinase was observed. These findings highlight the potential application of strains with anti-botrytis activity as sustainable alternatives to chemical defence for the control of this pathogen.

## 1. Introduction

In the contemporary agricultural landscape, sustainable crop management plays an increasingly critical role. Modern agriculture faces the challenge of ensuring high yields and quality while adapting to a rapidly evolving environmental scenario [1]. In response to this challenge, the adoption of alternative biocontrol strategies has emerged as a promising approach to protecting crops from pathogens and diseases, simultaneously reducing the use of harmful synthetic pesticides, risks of residues, and mitigating environmental impacts [2,3]. Grapevine cultivation spans approximately 7.6 million hectares worldwide, with Italy leading the forefront as the highest-output and largest wine producer [4]. This prominence underscores the importance of effective crop protection strategies, particularly against prevalent threats such as grey mould, caused by the fungus *Botrytis cinerea*. Grey mould poses a significant challenge in grapevine cultivation, leading to heavy reliance on plant defence agrochemicals to mitigate economic losses. However, this reliance presents substantial obstacles to achieving eco-sustainability objectives driven by the growing environmental awareness of producers and consumers. *B. cinerea* also compromises the quality of grapes and wine derived from infected vines, resulting in notable economic losses within the wine production sector [5]. Furthermore, as the effects of climate change increase, the challenges posed by *B. cinerea* on grapevines become more severe. Rising temperatures and heightened humidity levels, common in regions affected by climate change, create ideal conditions for the growth and spread of this pathogen [6]. Warmer temperatures speed up the life cycle of *B. cinerea*, leading to its rapid proliferation throughout vineyards. Additionally, increased humidity provides the necessary moisture for spore germination and infection, exacerbating the threat of this fungal disease [7,8]. Consequently, grape growers are confronted with heightened difficulties in managing and mitigating the impact of grey mould.

Traditionally, agrochemicals are predominantly used to control this pathogen, but are costly and less effective against a wide range of plant diseases. Additionally, failure to apply chemical protection during grey mould-favourable weather conditions can lead to substantial yield reduction and can compromise the aromatic profile of the wine [9,10]. Moreover, chemical control methods not only fall short of offering sustainable solutions in agriculture, but also carry the risk of exacerbating ecological issues such as the diffusion of pesticide resistance, for example, to benzimidazole-based fungicides [11]. Hence, there is a pressing need to explore alternative methods for reducing losses and boosting grapevine productivity through the adoption of biological control strategies, which hold considerable promise in this regard [12]. The limitations of conventional chemical treatments in agriculture are increasingly evident, particularly in terms of their environmental impact, diminishing efficacy due to pathogen resistance, and the growing governmental push for more sustainable viticulture practices. Consequently, deepening our knowledge on biological control agents (BCAs) offers a promising outlook for sustainable agriculture as their use represents an effective and environmentally friendly alternative for managing fungal diseases in agricultural settings.

In light of these concerns, there is growing interest in natural products, including BCAs, such as yeasts, fungi, and bacteria, to promote sustainability throughout the wine supply chain. Numerous studies have demonstrated the ability of various microorganisms with BCA activity to suppress crop diseases. Furthermore, BCAs offer advantages over synthetic fungicides, including having minimal impact on consumer health and the environment, making them integral to anti-resistance strategies [13]. In this sense, the research on new BCAs aligns with the guidelines of the EU and its member states, which advocate for reducing chemical inputs in agriculture through the use of greener technologies. In this context, the use of these bacteria is well suited, especially as grapevine pathogens become increasingly resistant to active substances over time, complicating control efforts.

However, despite their potential, it is crucial to acknowledge that the efficacy of BCAs against *B. cinerea* may vary. Thus, targeted screening efforts are essential to identify microbial strains with superior antagonistic capabilities. The selection and development of more effective biocontrol agents tailored to the specific environmental and agronomic conditions of *Vitis vinifera* cultivation can improve the overall efficacy of biological control strategies [14]. In this sense, certain *Burkholderia* spp. are BCA bacteria reported to produce positive effects in horticultural crops, including grapes, able to stimulate the growth of inoculated plants, and enhance plant adaptation to environmental stresses [15,16,17,18]. Another promising BCA bacteria species are *Pseudomonas* spp., which, for the non-pathogenic species [19], have already shown the ability to control *B. cinerea* on in vitro grapevine grown-plantlets [20], reducing also grey mould incidence by triggering defence-related genes in Chardonnay vineyards [21]. Additionally, there have been reports of *Pseudomonas fluorescens* strains diminishing disease in plant tissues by triggering a defence mechanism (induced systemic resistance—ISR), thereby bolstering grapevine resilience against subsequent infections from *B. cinerea* [22,23]. *Acinetobacter lwoffii* was reported as being capable of triggering resistance against *B. cinerea*, via induced systemic resistance mechanisms and up-regulation of key defence proteins [24,25,26].

In this context, this study evaluated the effectiveness of three putative BCA bacterial strains, previously selected for their in vitro efficacy against *B. cinerea* conidia germination and antifungal activity as potential BCAs in mitigating the effects of grey mould on grapes. Notably, this is the first time they have been tested on the ‘Glera’ cultivar. This case study, part of broader research that will serve as a basis for future experiments under various conditions (cultivars, environments, etc.), represents a preliminary investigation into the survival of these microorganisms in the carposphere. The study assesses their effectiveness in improving plant yield and must parameters, as well as their ability to reduce the incidence of *B. cinerea* at harvest. To investigate the local and systemic response of grapevine to grey mould colonization, the expression pattern of four defence-related genes selected from three different functional groups (Pathogenesis-related protein -PR-, glucanase, and chitinase) was analysed by RT qPCR.

## 2. Materials and Methods

### 2.1. Microorganisms

The BCA microorganisms tested in this study (*Paraburkholderia* sp. strain CRV74, *Pseudomonas* sp. strain CRV21, and *Acinetobacter* sp. strain CRV19) were isolated from Glera grapes during previous studies [27]. In brief, these strains were selected from among 80 isolates as their supernatants exhibited superior activity against *B. cinerea* strain DSM 5145 (obtained from the Leibniz Institute DSMZ—Deutsche Sammlung von Mikroorganismen und Zellkulturen Collection, Germany). In particular, these strains were selected for their in vitro efficacy against conidia germination and antifungal activity, which was assessed following protocols reported in the literature [28,29]. Strains were cryopreserved at −80 °C in Nutrient Broth (Merck, Darmstadt, Germany) supplemented with 50% *v*/*v* glycerol (Merck, Germany) until the moment of use. For the different analyses, strains were revived in Nutrient Broth at 30 °C for 48 h and then streaked twice on Nutrient Agar (Merck, Germany) at 30 °C for 48 h to ensure purity. To perform a preliminary identification, their DNA was extracted using the MagAttract HMW DNA kit (Qiagen, Germany), then sequencing of the amplicons of the V1-V3 region of the 16S rRNA gene, obtained via the primer pair P1 (5′-GCGGCGTGCCTAATACATGC-3′) and P4 (5′-ATCTACGCATTTCACCGCTAC-3′) [30] occurred. Using the BLASTn suite (https://blast.ncbi.nlm.nih.gov/Blast.cgi?PROGRAM=blastn&PAGE_TYPE=BlastSearch&BLAST_SPEC=&LINK_LOC=blasttab&LAST_PAGE=tblastn, accessed on 3 June 2024), the most related sequence in the NCBI nucleotide sequence database was then determined [31]; a further phylogenetic analysis aligning the sequences with the reference genomes reported on NCBI for the most related species within the genus identified using TYGS [32] (Table 1) was performed, constructing then the phylogenetic tree using MAFFT [33] and FastTree [34], which was lastly plotted using FigTree. To test their efficacy against *B. cinerea*, pure colonies of each strain were inoculated in Nutrient Broth kept in agitation at 30 °C for 24 h. Bacterial cultures were then collected by centrifuging the growth media at 4500× *g* at 4 °C for 15 min. Pelleted cells were resuspended in sterile saline-peptone water (9 g/L NaCl, 1 g/L bacteriological peptone) to prepare standardized suspensions with an OD_600_ = 0.1 (corresponding to 10^7^ CFU/mL). Then, 50 mL of these suspensions were sprayed on the grapes of each treated plant, to achieve a potential treatment spraying of 200 hL/ha. The persistence of inoculated strains was assessed during the evaluation of grey mould symptoms on grapes through species-specific PCR. To this end, 50 g of randomly collected grape berries were homogenized. From these, a 2 mL sample aliquot was taken for DNA extraction using the DNeasy PowerFood Microbial Kit (Qiagen, Hilden, Germany). Amplification was carried out using species-specific primers and protocols reported in the literature for *Burkholderia* spp. [35], *Pseudomonas* spp. [36], and *Acinetobacter* spp. [37], with amplifications performed using the MiniAmp Thermal Cycler (Thermofisher, Waltham, MA, USA). The inoculum of *B. cinerea* was obtained by culturing the mould on Potato Dextrose Agar (PDA) (Merck, Germany) at 20 °C for 20 days. The surface of the plates was then scraped to collect spores that were resuspended in in Potato Dextrose Broth (PDB) (Merck, Germany). Conidial concentrations were assessed using a Bürker counting chamber (Merck, Darmstadt, Germany), and the final density was adjusted to 10^5^ conidia/mL. After 3 h of incubation at 20 °C and 150 rpm, 50 mL of PDB containing germinated spores were sprayed on each plant for inoculation.

### 2.2. Experimental Set Up

The trial was conducted during the 2023 growing season in a greenhouse scale using 4-years-old *Vitis vinifera* ‘Glera’ vines grafted onto 1103 P rootstock. The treatments were NT: not-treated control plants, BP: *Paraburkholderia* sp. strain CRV74 treated plants, PF: *Pseudomonas* sp. strain CRV21 treated plants, AL: *Acinetobacter* sp. strain CRV19 treated plants (five replicates each). 

**Figure 1:**
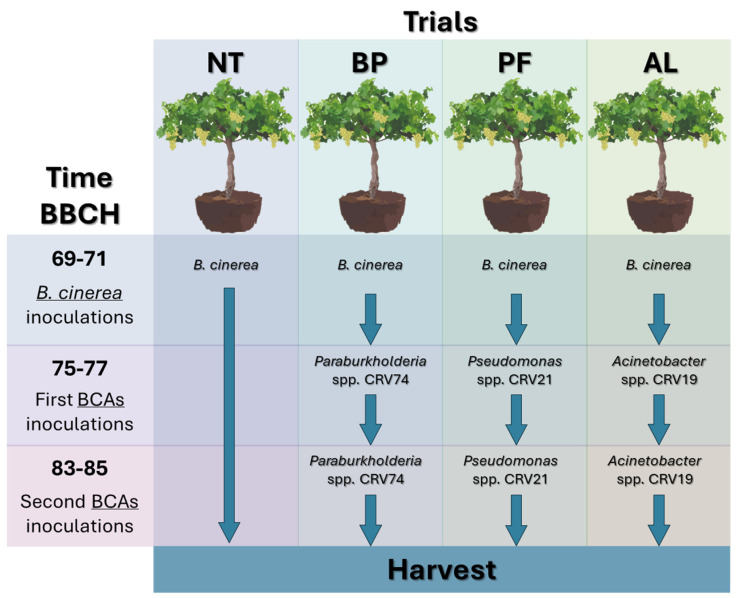
Plant experimental design. NT: not-treated control plants, BP: *Paraburkholderia* sp. strain CRV74 treated plants, PF: *Pseudomonas* sp. strain CRV21 treated plants, AL: *Acinetobacter* sp. strain CRV19 treated plants. *B. cinerea* inoculations were performed at BBCH 69-71, followed by two inoculums of BCAs at BBCH: 75-77, 83-85.

The artificial inoculation of grey mould (*Botrytis cinerea*) was performed at BBCH 69-71 (Biologische Bundesanstalt, Bundessortenamt and CHemical industry–phenological stages), replicating what naturally occurs in open field conditions. The BCAs were inoculated twice, at BBCH 75-77 and at BBCH 83-85 (Figure 1). Plants followed the same micro irrigation protocol, not fertilized, and plant agrochemicals (copper and sulphur), were applied in the same quantity across the treatments only to avoid possible infections of downy (*Plasmopara viticola*) and powdery (*Erysiphe necator*) mildews.

### 2.3. Evaluation of Grey Mould Symptoms on Grapes, and Yield

The effectiveness in controlling grey mould symptoms was evaluated visually by inspecting the grapes for *B. cinereal* symptoms, comparing treated and control grapes. Disease incidence was recorded as the percentage (%) of the infected grapes per each plant. The symptoms included greyish fuzzy mould growth on the surface of the berries, the presence of soft, rotting areas as well as discoloration and shrivelling of berries [38].

Furthermore, the total commercial yield of the plants was assessed. Grapes were hand-picked on the same day and weighed using a digital dynamometer (Sinergica soluzioni S.r.l., Milan, Italy) [39]. Total soluble solids content (TSS), expressed as °Brix, was measured with an automatic refractometer (Atago PR32, Tokyo, Japan). Total acidity of berries expressed both as pH and titratable acidity (i.e., g/L of tartaric acid) were measured using an automatic titrator (Crison Micro TT 2022, Alella, Spain) by titration with 0.1 N NaOH solution [40].

### 2.4. Gene Expression Analysis by Quantitative Real-Time PCR (qRT-PCR)

Grapevine berry peel samples were collected when the *Botrytis*-symptoms reached at least 50% in untreated vines. Twenty-five infected berries per replicate were collected from treated and untreated grapevines, and immediately frozen in liquid N_2_ and stored at −80 °C until RNA extraction. Total RNA extraction was performed using the Spectrum Plant total RNA kit (Merck, Germany), followed by quantification using NanoDrop 8000 (Thermo Fisher, USA) [41]. After treating the RNA with DNAse I (Invitrogen, Waltham, MA, USA), the first-strand cDNA synthesis was carried out using Superscript III (Invitrogen, USA) and oligo-dT primer from 1.0 μg of total RNA. Quantitative real-time PCR was carried out using Platinum SYBR Green qPCR SuperMix-UDG (Invitrogen, USA) and specific primer pairs (as detailed in Table 2) on the LightCycler 480 Instrument II, utilizing its SV1.5.0 software (Roche Diagnostics, Mannheim, Germany). PCR conditions included an initial step of 50 °C for 2 min and 95 °C for 2 min, followed by 40 cycles of 95 °C for 15 s and 60 °C for 1 min [42]. Each sample underwent analysis in five technical replicates, and dissociation curves were examined to confirm amplification specificity. Relative gene expression was determined using the Pfaffl equation [43], with berries from control plants serving as calibrators. Actin was chosen as the constitutive gene for normalization due to its unaffected expression by treatments, with comparable results observed using actin primer pairs. The impact of each bacterial strain on grapevine defence response against *B. cinerea* infection was calculated as the ratio between expression levels in symptomatic berries of treated vs. untreated plants, with a 1.5-fold threshold used to identify enhanced expression. Mean expression and standard error values were calculated based on five replicates per sample.

### 2.5. Statistical Analyses

Statistical analysis on the collected data was performed using R version 4.1.2. Significant differences between treatment means were assessed using one-way ANOVA test (*p* ≤ 0.05), and performing Tukey HSD test (*p* ≤ 0.05) as a post-hoc test. The validation of the linear model hypotheses was conducted through the assessment of the normal distribution of values and residuals. The normal distribution was evaluated using the Shapiro–Wilk test. For all treatments, the calculated *p*-value exceeded the critical *p*-value (0.05); thus, the null hypothesis (H0) was always accepted. The normality of the residual’s distribution was assessed using the Kolmogorov–Smirnov test. For all treatments, the tabulated D value (0.265) was greater than the calculated D value; hence, the null hypothesis (H0) was consistently accepted.

## 3. Results

### 3.1. Strains

**Figure 2:**
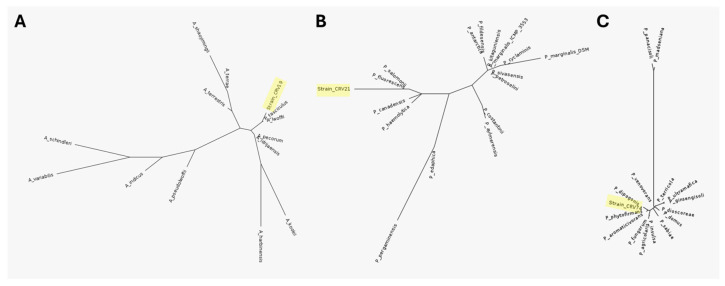
Phylogenetic trees for strain CRV19 (**A**), CRV21 (**B**), and CRV74 (**C**).

After sequencing of the V1–V4 region of the 16S rRNA gene, the most related sequence in the NCBI nucleotide sequence database was determined using the BLASTn suite for each strain (Table 3). For strain CRV19, the most related species resulted in *Acinetobacter lwoffi*; for strain CRV21, it resulted in *Pseudomonas fluorescens*; and for strain CRV74, it resulted in *Paraburkholderia phytofirmans*. However, after the phylogenetic analysis, it was not possible to obtain a unique identification of the strains. Indeed, for strain CRV19, the identification resulted in ambiguity between *A. lwoffi* and *Acinetobacter idrijanensis* due to identical 16S rRNA gene sequences (Figure 2A). It is worth noting that *A. idrijanensis* does not have a valid published name and may be a misdeposited reference strain. Similarly, for strain CRV74, the identification resulted in ambiguity between *P. phytofirmans*, *Paraburkholderia aromaticivorans*, and *Paraburkholderia dipogonis* due to identical sequences among these reference strains (Figure 2B). Lastly, strain CRV21 was misidentifiable between *P. fluorescens* and *Pseudomonas salomonii*, which also present identical sequences (Figure 2C). It should be emphasized that no matches with potential pathogenic species were found.

### 3.2. Harvest Yield and Must Parameters

**Figure 3:**
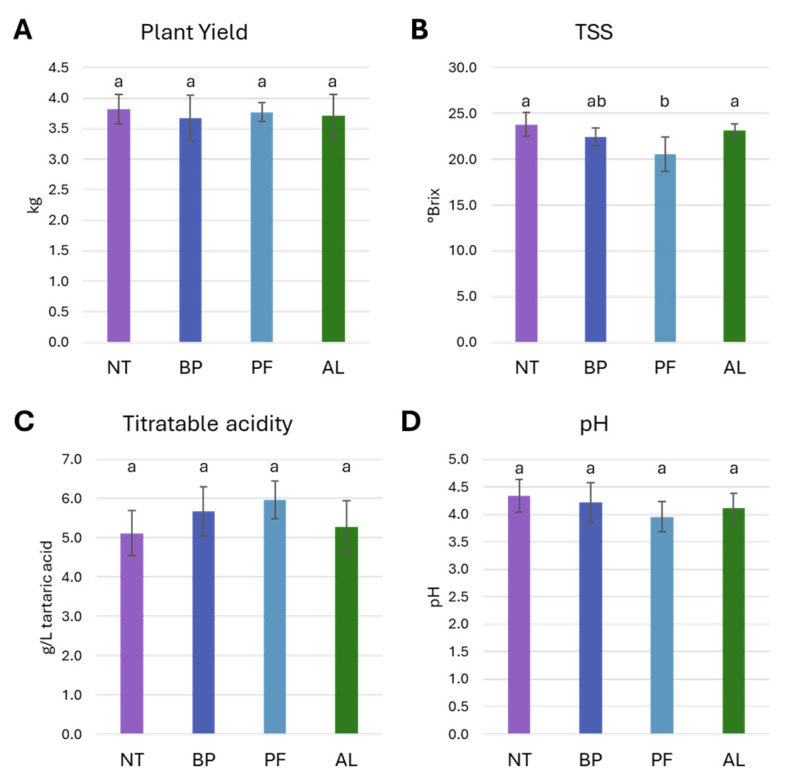
Effect of the application of *Paraburkholderia* sp. strain CRV74 (BP), *Pseudomonas* sp. strain CRV21 (PF), and *Acinetobacter* sp. strain CRV19 (AL) on yield and berry quality in comparison to untreated plants (NT). (**A**) Average plants yield, expressed in kilograms; (**B**) must total soluble solids (TSS), expressed as °Brix; (**C**) titratable acidity, expressed as g/L of tartaric acid and (**D**) pH values. Where present, different letters indicate significant differences identified through Tukey post hoc test (*p* < 0.05). Bars denote the standard deviation.

In comparison to the control group (NT), which yielded 3.8 ± 0.2 kg, no significant differences were observed in the overall production yield for plants treated with BP (3.7 ± 0.4 kg), PF (3.8 ± 0.2 kg), or AL (3.7 ± 0.4 kg) (Figure 3A). However, significant impact on TSS was noted. In fact, PF application resulted in a significant decrease of TSS (20.5 ± 1.8 °Brix) in comparison to the control (NT: 23.8 ± 1.3 °Brix) and the other strains (AL: 23.1 ± 0.8 °Brix; BP: 22.4 ± 1.0 °Brix) (Figure 3B). Regarding acidity, the highest levels were observed in the PF treatment (4.0 ± 0.3 pH; 6.0 ± 0.58 g/L) compared to the NT control where lower acidity was observed (4.3 ± 0.3 pH; 5.12 ± 0.6 g/L), with intermediate values for BP (4.2 ± 0.4 pH; 5.7 ± 0.6 g/L) and AL (4.1 ± 0.3 pH; 5.3 ± 0.7 g/L); however, differences were not statistically significant (Figure 3C,D).

### 3.3. Symptoms Evaluation

**Figure 4:**
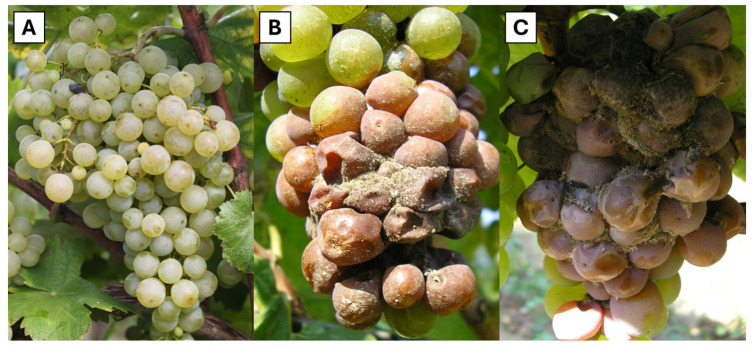
Image (**A**): PF-treated grape, (**B**,**C**): untreated grapes (% of infection: 55 to 65). The photos were taken at the same BBCH (87), showing that the development of grey mould was delayed on the PF treatment. Botrytis had already emerged from the infected berries (sporulation stage).

**Figure 5:**
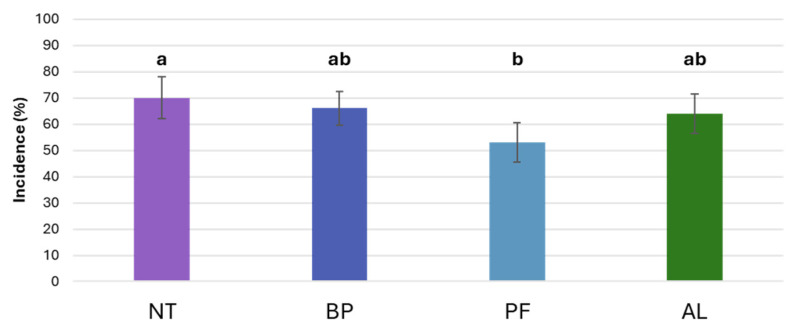
Incidence of symptomatic berries of ‘Glera’ in vines treated with *Paraburkholderia* sp. strain CRV74 (BP), *Pseudomonas* sp. strain CRV21 (PF), and *Acinetobacter* sp. strain CRV19 (AL), and in untreated plants (NT). Letters indicate significant differences identified through Tukey post hoc test (*p* < 0.05). Bars denote the standard deviation.

After analysing the treatment-induced variances (Figure 4), significant variations (*p* < 0.05) in *B. cinerea* symptoms were observed. Specifically, compared to untreated control plants (NT) where the incidence of grey mould stood at 70.0 ± 7.9%, similar outcomes were observed for BP (66.0 ± 6.5%) and AL (64.0 ± 7.4%). In contrast, a significant reduction in symptoms was observed in grapes treated with PF (53.0 ± 7.6%) (Figure 5).

### 3.4. Modulation of Defence-Related Genes

**Figure 6:**
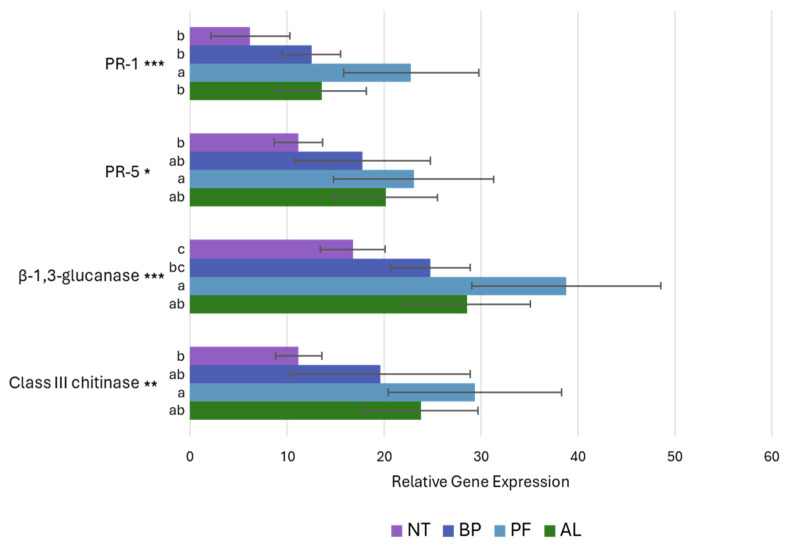
Relative gene expression of PR-1, PR-5, β-1,3-glucanase, and class III chitinase in relation to the treatment with *Paraburkholderia* sp. strain CRV74 (BP), *Pseudomonas* sp. strain CRV21 (PF), and *Acinetobacter* sp. strain CRV19 (AL) compared to untreated plants (NT). Statistical significance is reported as follows: *** *p* < 0.001, ** *p* < 0.01, * *p* < 0.05. Letters indicate significant differences identified through Tukey post hoc test. Bars denote the standard deviation.

Considering the modulation effects on defence-related genes PR-1, PR-5, β-1,3-glucanase, and class III chitinase, an upregulation effect induced by BCAs was consistently identified in each treatment (Figure 6). However, only PF exhibited statistically significative upregulation of all defence genes compared to the NT control. This indicates that PF effectively stimulated the upregulation of these genes, bolstering the plant’s defence mechanisms against grey mould infection. BP and AL also resulted in increased expression levels of defence genes compared to the untreated group, albeit with effects inferior to those of PF. Specifically, for these microorganisms, the only significant difference observed compared to the NT control was noted in the upregulation of the β-1,3-glucanase gene following treatment with AL.

## 4. Discussion

*Botrytis cinerea* is the causative agent of grey mould in over 500 dicot plants, including economically important crops like grapevines, by attacking various tissues such as stems, leaves, and fruits [45]. Estimating the economic impact of *B. cinerea* is difficult due to its wide host range, but annual losses are estimated between USD 10 billion and USD 100 billion globally [46]. Controlling this pathogen necessitates exploring alternatives to synthetic agrochemicals to minimize environmental harm [47,48]. Biological control agents (BCAs) like *Paraburkholderia* spp., *Pseudomonas* spp., and *Acinetobacter* spp. have shown effectiveness against grey mould. This study aims to evaluate the biocontrol efficacy of three strains of these BCAs on the grapevine variety ‘Glera’, which is important in the Italian grapevine industry.

As a first step of this study, a preliminary identification of the three selected bacterial strains was performed. At this stage of the research, the identification was performed by sequencing the V1–V4 region of the 16S rRNA gene due to its relatively low cost, ease of use, and the extensive database of 16S rRNA sequences available for comparison. However, in this case, 16S rRNA sequencing was not sufficient to obtain a unique identification of our strains due to the high similarity of our sequences to several reference strains in the BLAST database. For this reason, in future research, analyses based on whole genome sequencing will be conducted, not only to achieve a taxonomic identification but also to gain a deeper understanding of the resistance mechanisms induced, thanks to the possibility of conducting an in-depth genetic characterization of the strains. 

The incidence of grey mould symptoms, yield, and the expression levels of key genes encoding proteins or enzymes involved in various defence-related metabolic pathways were then evaluated in BCA-treated grapes in comparison to non-treated controls. When assessing overall grapevine yield, no statistically significant differences were found among the treatments. However, a significant difference was noted in the °Brix values, especially within the NT untreated control group (where grapes also showed more pronounced botrytization) compared to the PF treatment. This phenomenon can be attributed to grape rot in various ways. *B. cinerea* infiltrates grape skins, triggering dehydration and leading to increased sugar concentration within the fruit. Simultaneously, the grapevine responds to infection by enhancing photosynthesis rates, thereby increasing sugar production [49,50]. Furthermore, lower TSS and higher acidity are also characteristic of unripe berries. The vulnerability of grape clusters to *Botrytis* rot steadily rises from the veraison stage to ripening. [51]. Since *B. cinerea* produces ethylene during the infection process, which is a hormone that promotes the ripening of berries, the higher TSS and lower acidity in non-treated grapes may be related to a more advanced ripening stage, induced by the higher incidence of grey mould of infected berries and the consequent exposure to ethylene produced by the pathogen. In this context, PF musts reached optimal technological levels of sugars and acidity, both crucial parameters for wine quality, especially considering the perspective of climate change, where rising temperatures significantly impact them [52,53,54]; these results were further confirmed by other works reported in the literature [55]. Furthermore, in comparison to control plants, the application of *Pseudomonas* sp. strain CRV21 was associated with a significant reduction in symptoms linked to *B. cinerea*; while considering BP and AL trials, although symptoms were reduced, these were not significant. Similar results with grey mould symptom reduction from 43% to 20% resulting from the application of *P. fluorescens* have been reported in the literature by other authors [23].

PF treatment efficacy was therefore reflected also in the modulation of plant defence gene expression (PR-1, PR-5, β-1,3-glucanase, and class III chitinase), which showed significant upregulation, which was also reported in other studies [22,24]. The ability of BCA microbes like *Pseudomonas* spp. (e.g., *P. fluorescens*) to induce the up-regulation of these genes, which play distinct roles in fortifying the plant’s defences [56,57], is crucial for enhancing plant health and resilience [58]. PR-1 and PR-5 are proteins involved in systemic acquired resistance (SAR), an advanced defence mechanism that primes the entire plant against invading pathogens. By triggering the synthesis of these pathogenesis-related proteins, *P. fluorescens* could function as an effective priming of the immune response, impeding pathogen proliferation, and safeguarding the plant from systemic infections [59]. Similarly, the upregulation of β-1,3-glucanase enhances the plant’s ability to dismantle fungal cell walls, a crucial defence system against fungal intrusion. The stimulation of enzymatic activity through the use of BCA bacteria can thus act as a frontline defence, impeding fungal colonization, and averting potential damage to the plant [60]. Similarly, class III chitinases provide another layer of defence against pathogens. Chitinase enzymes break down chitin, a structural component of fungal cell walls and insect exoskeletons. The ability to stimulate their production by *Pseudomonas* spp. can thus provide the plant with tools to degrade chitin, thereby thwarting fungal growth and infestation [22,61]. Overall, the coordinated upregulation of these defence proteins primes the plant’s immune system, enhancing its resilience against diverse pathogens. This priming effect not only enables a swift and robust response to immediate threats but also establishes a lasting defence readiness, ensuring the plant’s long-term health and productivity, in contrast to phytochemical treatments whose efficacy diminishes over time [62]. Thus, the symbiotic interaction between plants and beneficial microbial strains like *Pseudomonas* sp. strain CRV21 tested in this work exemplifies nature’s ingenious strategies for bolstering plant defence mechanisms.

In conclusion, the obtained data suggested the importance of selection of strains with BCA functions, that hold promise as an alternative strategy in combating *B. cinerea* on grapevines. In particular, *Pseudomonas* sp. strain CRV21 showed a significant ability to upregulate key defence proteins, and showed potential applications for fighting this fungal pathogen, reducing environmental impact, and offering prolonged efficacy compared to conventional treatments, as evidenced by symptom evaluation. These findings offer promising avenues for sustainable disease management in agriculture.

## Figures and Tables

**Table 1:** List of reference genomes with their accession numbers used for phylogenetic analysis.

Preferred Name	Deposit	Assembly Accession	Notes
*Acinetobacter terrae*	ANC 4282	GCA_013004375	
*Acinetobacter pseudolwoffii*	ANC 5044	GCA_002803605	
*Acinetobacter kookii*	JCM18512	GCA_039543765	
*Acinetobacter mesopotamicus’*	DSM 26953	GCA_011058205	Not a valid published name
*Acinetobacter shaoyimingii*	323-1T	GCA_011578045	
*Prolinoborus fasciculus*	CIP 103579	GCA_900322255	
*Acinetobacter variabilis*	NIPH 2171	GCA_000369625	
*Acinetobacter lwoffii*	NCTC 5866	GCA_000487975	
*Acinetobacter schindleri*	CIP 107287	GCA_000368625	
*Acinetobacter indicus*	CIP 110367	GCA_000488255	
*Acinetobacter harbinensis*	HITLi 7	GCA_000816495	
*‘Acinetobacter pecorum’*	Sa1BUA6	GCF_014837015	Not a valid published name
*Acinetobacter terrestris*	ANC 4471 T	GCA_004331155	
*‘Acinetobacter idrijaensis’*	MII	GCA_000761495	Not a valid published name
*Paraburkholderia phytofirmans*	PsJN	GCA_000020125	
*Paraburkholderia dipogonis*	ICMP 19430	GCF_004402975	
*Paraburkholderia dioscoreae*	Msb3T	GCF_902459535	
*Paraburkholderia xenovorans*	LB400	GCA_000013645	
*Paraburkholderia aromaticivorans*	BN5	GCA_002278075	
*Paraburkholderia ultramafica*	LMG 28614	GCA_902859915	
*Paraburkholderia ginsengisoli*	NBRC 100965	GCA_000739735	
*Paraburkholderia panacisoli*	DCY113	GCA_008369935	
*Paraburkholderia terricola*	LMG 20594	GCA_900142195	
*Paraburkholderia insulsa*	LMG 28183	GCA_003002115	Reclassified as *P. fungorum*
*Paraburkholderia fungorum*	NBRC 102489	GCA_000685055	
*Paraburkholderia agricolaris*	BaQS159	GCF_009455635	
*Paraburkholderia madseniana*	RP11	GCA_009690905	
*Paraburkholderia domus*	LMG 31832	GCA_905220705	
*Paraburkholderia phenazinia*	LMG 2247	GCA_900100735	No Match
*Paraburkholderia sabiae*	LMG24235	GCF_904848645	
*Pseudomonas fluorescens*	DSM 50090	GCA_001269845	
*Pseudomonas salomonii*	LMG 22120	GCA_001730645	
*Pseudomonas edaphica*	RD25	GCA_005863185	
*Pseudomonas antarctica*	LMG 22709	GCF_900103795	
*Pseudomonas kitaguniensis*	MAFF 212408T	GCF_009296165	
*Pseudomonas costantinii*	LMG 22119	GCF_001870435	
*Pseudomonas cyclaminis*	MAFF 301449T	GCA_015163715	
*Pseudomonas sivasensis*	P7	GCA_013778505	
*Pseudomonas marginalis*	DSM 13124	GCA_007858155	
*Pseudomonas aylmerensis*	S1E40	GCA_001702265	
*Pseudomonas marginalis*	ICMP 3553	GCA_001645105	
*Pseudomonas petroselini*	MAFF 311094	GCA_021166635	
*Pseudomonas canadensis*	2-92	GCF_000503215	
*Pseudomonas pergaminensis*	1008T	GCF_024112395	
*Pseudomonas haemolytica*	DSM 108987T	GCF_009659625	
*Pseudomonas fildesensis*	KG01	GCA_001050345	

**Table 2:** List of primers used for the gene expression analyses in grapevine berries, cv. Glera, and NCBI accession numbers. References: PR-1 and PR-5. β-1,3-glucanase and class III chitinase. Housekeeping gene: Actin.

Genes	Primer Pairs	Accession Number *	Reference
PR-1	Forward: ACTTGTGGGTGGGGGAGAA	AJ536326	[3]
	Reverse: TGTTGCATTGAACCCTAGCG		
PR-5	Forward: GACGGGCTGGTCAGGTC	TC118300	[3]
	Reverse: CGCCGTTGCACTCTACCT		
β-1,3-glucanase	Forward: TGCTGTTTACTCGGCACTTG	AJ277900	[44]
	Reverse: CTGGGGATTTCCTGTTCTCA		
class III chitinase	Forward: AAACTTATCAGCGCCTGGAA	DQ406693	[44]
	Reverse: ACCTCCATACTTGGGGGAAG		
Actin	Forward: TCCTTGCCTTGCGTCATCTAT	TC134791	[42]
	Reverse: CACCAATCACTCTCCTGCTACAA		

* Accession numbers from NCBI Gene Bank (www.ncbi.nlm.nih.gov) or TIGR Grape database v7.0 (https://www.genoscope.cns.fr/cgi-bin/ggb/vitis/12X/gbrowse/vitis/, accessed on 3 June 2024).

**Table 3:** 16S ribosomal RNA identification of isolates.

Strain	Accession Number	Seq. Length (bp)	Species	Reference Strain WGS	Identity (%)	Query Cover (%)
CRV19	PP927971.1	496	*Acinetobacter lwoffi*	GCA_019343495.1	99.20	100
CRV21	PP927970.1	517	*Pseudomonas fluorescens*	GCA_900215245.1	98.84	100
CRV74	PP927969.1	518	*Paraburkholderia phytofirmans*	GCA_000020125.1	99.23	100

## Data Availability

The raw data supporting the conclusions of this article will be made available by the authors on request.
